# The serum level of D-Dimer is not suitable for distinguishing between prosthetic joint infection and aseptic loosening

**DOI:** 10.1186/s13018-019-1461-x

**Published:** 2019-11-29

**Authors:** Jincheng Huang, Yongchao Zhang, Zhen Wang, Yonghui Dong, Yongqiang Zhao, Jia Zheng, Hongkai Lian, Yi Jin

**Affiliations:** 1 Department of Orthopedics, Henan Provincial People’s Hospital, People’s Hospital of Zhengzhou University, People’s Hospital of Henan University, Zhengzhou, 450003 China; 2 Department of Orthopedics, Zhengzhou Central Hospital, Zhengzhou University, Zhengzhou, 450007 China

**Keywords:** Prosthetic joint infection, D-Dimer, ESR, CRP, Diagnosis

## Abstract

**Background:**

To evaluate the meaning of serum CRP, ESR, and D-Dimer in the diagnosis of prosthetic joint infection.

**Methods:**

In a retrospective study, 101 patients presented with osteoarthritis, PJI, and aseptic loosening were divided into three groups according to the type of operation they received in our department from June 2016 to December 2018: group A, 44 patients treated with primary arthroplasty; group B, 31 PJI patients treated with resection arthroplasty and spacer insertion surgery; group C, 26 aseptic loosening patients treated with revision arthroplasty. Data such as gender, age, preoperative serum CRP, ESR, and D-Dimer level were compared among the three different groups.

**Results:**

There are no statistically significant differences when comparing general data such as gender and age in patients from the three different groups. However, Serum CRP level in group B (43.49 ± 10.00 mg/L) is significantly higher than in group A (2.97 ± 0.75 mg/L) and C (4.80 ± 1.26 mg/L). Serum ESR level in group B (49.84 ± 5.48 μg/L) is significantly higher than those in group A (15.28 ± 2.63 μg/L) and C (22.50 ± 3.47 μg/L). Serum D-Dimer level in group B (1.58 ± 0.17 μg/L) is significantly higher than that in group A (0.51 ± 0.50 μg/L), but similar with group C (1.22 ± 0.29 μg/L). There are no statistically significant differences when compared with sensitivity and specificity of CRP, ESR, and D-Dimer in the diagnosis of PJI among patients from the three different groups when D-Dimer > 0.85 μg/L was set as the optimal threshold value for the diagnosis of PJI.

**Conclusion:**

D-Dimer is not a parameter to distinguish between aseptic loosening and PJI.

## Background

It remains a big challenge for the clinician to make an accurate prosthetic joint infection (PJI) diagnosis. Although serum CRP and ESR are the most commonly used serological markers in PJI diagnosis [1, 2], they did not perform well in some situations such as low-virulence organism infection [3] and indolent microorganism infection [4]. As a result, numerous serological markers for PJI have been evaluated in the past, including interleukin 6 (IL-6) [5], serum soluble intercellular adhesion molecule-1 (sICAM-1) [6], toll-like receptor(TLRs) [7], lipopolysaccharide-binding protein (LBP) [8, 9], myeloid-related protein14 (MRP-14) [10], and soluble urokinase plasminogen activation receptor (su-PAR) [11]. Although some of them exerted exciting performance in PJI diagnosis, it is still not realistic to popularize them in clinical practice especially in primary hospitals in the next a few years due to high expense and their reliability.

Recently, serum D-Dimer, one of the most commonly checked serological markers in joint surgery departments, was demonstrated to be a promising marker for the diagnosis of PJI [12] and timing of reimplantation [13]. However, there are only a few papers related to the relationship between serum D-Dimer and PJI in the literature, so, we want to determine the meaning of serum CRP, ESR, and D-Dimer in the diagnosis of PJI and evaluate whether D-Dimer performs better than CRP and ESR.

## Methods

We retrospectively analyzed clinical data of 101 patients presented with primary osteoarthritis or secondary to hip congenital, PJI, and aseptic loosening in our department from June 2016 to December 2018. Exclusion criteria include the following: patients with 1, any type of skin ulcer or hematoma; 2, a history of recent dislocation or trauma (within 2 weeks); 3, visible ecchymosis; 4, a prosthetic heart valve; 5, a history of hyper-coagulation disorder; 6, systemic inflammatory disease (such as rheumatoid arthritis, psoriasis, systemic lupus erythematosus (SLE), polymyalgia rheumatica, hepatitis B and C, inflammatory bowel disease (IBD), sarcoidosis, gout, myelodysplastic syndrome, lymphocytic leukemia, and multiple myeloma); 7, tumor. Patients enrolled in this study were grouped as follows: group A: 44 patients treated with primary arthroplasty; group B: 31 PJI patients treated with resection arthroplasty and spacer insertion surgery; group C: 26 aseptic loosening patients treated with revision arthroplasty. None of the patients in groups A through C was thought to have concurrent infections.

Data such as gender, age, preoperative serum CRP, ESR, and D-Dimer level were recorded in patients from the three different groups. PJI was defined using the MSIS criteria, in which the limit values of CRP and ESR for detection of PJI is above 10 mg/L and 30 mm/h respectively [14]. Aseptic loosening was defined with the following criteria: 1, pain in the thigh or hip region, knee pain; 2, radiological symptoms of loosening (disintegration of prosthesis components with the bone, displaced components of the prosthesis, circumferential radiolucent line); 3, cannot be defined as PJI. Patient demographics are presented in Table 1.

**Table 1:** Comparison of the general data among patients from three different groups

Group	Age	Gender	
		Male	Female
A	65.64 ± 1.05	14	19
B	64.94 ± 2.70	11	13
C	69.27 ± 1.55	9	14
Statistic	*F* = 1.966	*χ*^2^ = 0.216	
	*P* = 0.145	*P* = 0.898	

## Statistical analysis

Quantitative data were expressed as mean ± standard deviation; single factor analysis of variance was used to compare difference among multiple groups. Chi-square test (*χ*^2^) was used to compare the counting data among groups. *P* value less than 0.05 was considered significant difference. All statistical analyses were performed using IBM SPSS Statistics (version 19, IBM SPSS Software).

## Results

**Fig. 1:**
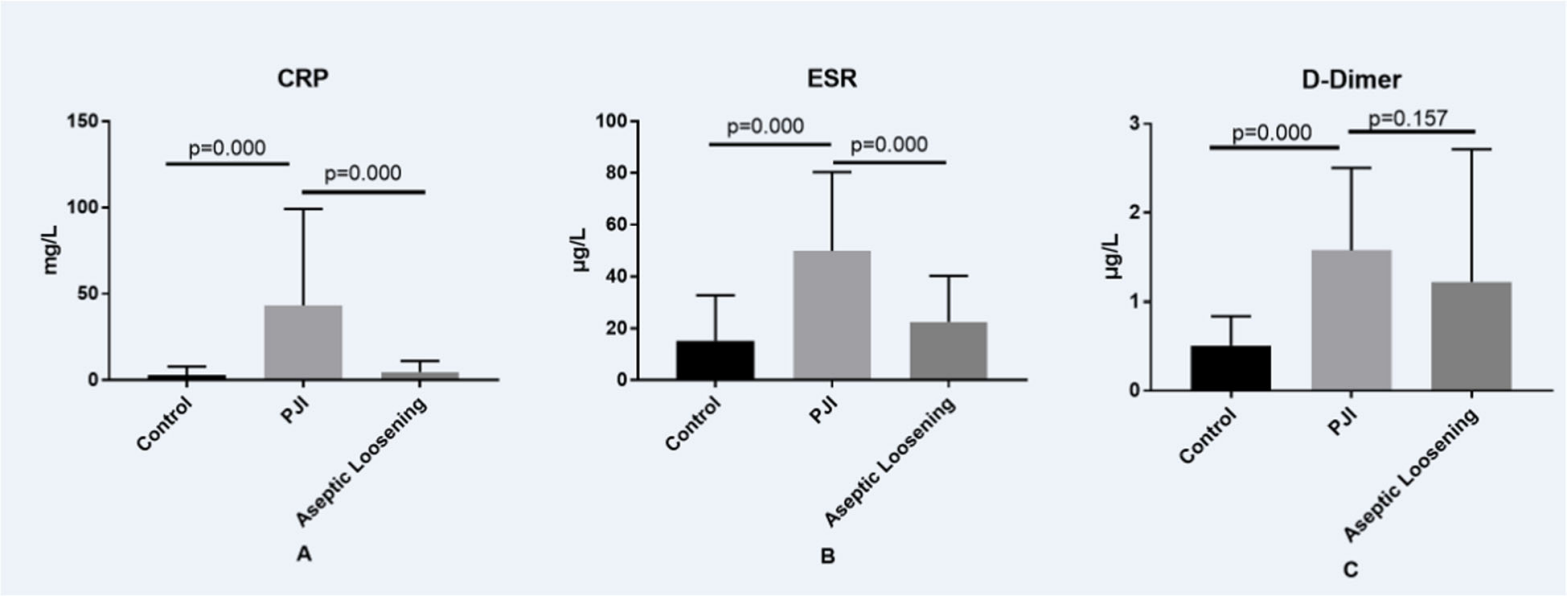
Expression of serum CRP, ESR and D-Dimer in patients from the three different groups

As shown in Fig. 1, serum CRP level in group B (43.49 ± 10.00 mg/L) is significantly higher than those in group A (2.97 ± 0.75 mg/L) and C (4.80 ± 1.26 mg/L), and serum ESR level in group B (49.84 ± 5.48 μg/L) is also significantly higher than those in group A (15.28 ± 2.63 μg/L) and C (22.50 ± 3.47 μg/L). However, different from CRP and ESR, serum D-Dimer level in group B (1.58 ± 0.17 μg/L) is only significantly higher than that in group A (0.51 ± 0.50 μg/L), but similar with that in group C (1.22 ± 0.29 μg/L).

As paper published by Yong et al. [12] and Alisina et al. [13] showed that serum D-Dimer is a promising serological marker for the diagnosis of PJI especially when 0.85 μg/L was determined as the optimal threshold value, so, we decided to compare the sensitivity and specificity of serum CRP (> 10 mg/L), ESR (> 30 mm/h) and D-Dimer (> 0.85 μg/L) in the diagnosis of PJI. As showed in Tables 2 and 3, the sensitivity of serum CRP, ESR and D-Dimer is 0.68, 0.74 and 0.71 respectively; the specificity of serum CRP, ESR, and D-Dimer is 0.93, 0.87, and 0.80 respectively, there are no statistically significant differences when compared with sensitivity and specificity of CRP, ESR, and D-Dimer in the diagnosis of PJI among patients from the three different groups.

**Table 2:** Comparison of the sensitivity of CRP, ESR, D-Dimer in diagnosis of PJI among patients from three different groups

	True positive	False negative	Sensitivity
CRP (> 10 mg/L)	21	10	0.68
ESR (> 30 mm/h)	23	8	0.74
D-Dimer (> 0.85 μg/L)	22	9	0.71

*F* = 0.313, *P* = 0.855. There are no statistically significant differences when compared sensitivity of CRP, ESR, D-Dimer in diagnosis of PJI among patients from three different groups

**Table 3:** Comparison of the specificity of CRP, ESR, D-Dimer in diagnosis of PJI among patients from three different groups

	True negative	False positive	Specificity
CRP (> 10 mg/L)	65	5	0.93
ESR (> 30 mm/h)	61	9	0.87
D-Dimer (> 0.85 μg/L)	56	14	0.80

*F* = 5.027, *P* = 0.081. There are no statistically significant differences when compared specificity of CRP, ESR, D-Dimer in diagnosis of PJI among patients from three different groups

## Discussion

Despite the availability of various auxiliary tests, the accurate diagnosis of PJI continues to be challenging. Due to the convenience, non-invasion and rapidity of serological examination, it is always the first choice for the clinicians to make a PJI diagnosis. Although lots of efforts have been made to improve the accuracy of serological markers in PJI diagnosis, there is still lack of a universally acknowledged serological marker.

Although CRP and ESR are the first-line screening serological markers for PJI diagnosis, papers have different conclusions on their roles in PJI diagnosis. Saleh et al. [15] demonstrated that until now, no other serum biomarkers have been demonstrated superior than CRP and ESR in PJI diagnosis. Berbari et al. [16] found that interleukin-6 is the better than CRP and ESR for PJI diagnosis and CRP is more accurate than ESR in predicting PJI. Spangehl et al. [17] found the sensitivity (SE) and specificity (SP) of CRP and ESR in PJI diagnosis is (SE 82%, SP 85%) and (SE 96%, SP 92%) respectively; combination of CRP and ESR is reliable for predicting the absence of infection. Perez et al. [18] found that CRP and ESR may not be accurate as diagnostic tools in PJI, particularly to identify low-virulent microorganisms (such as coagulase-negative staphylococci, *Bacillus* species, *Corynebacterium* species and *Propionibacterium* species) and chronic PJI. Akgün et al. [3] found that CRP alone is not an accurate screening tool for PJI and may have high false negative rates, especially when the causative organism has low virulence. So, it looks like that the type of infecting organism has great effect on the level of CRP and ESR, and CRP and ESR are not reliable for predicting low-virulent PJI. In this study, we found that the SE and SP of CRP and ESR in PJI diagnosis are (SE 68%, SP 93%) and (SE 74%, SP 87%) respectively, which to some extent implies both CRP and ESR could be used and have similar meaning for PJI diagnosis. But due to our limited samples (31 PJI patients), we cannot make a conclusion whether CRP or ESR is better for PJI diagnosis and CRP or ESR alone is sufficient for PJI diagnosis.

As both CRP and ESR have their limitations in PJI diagnosis and the Consensus document for the diagnosis of PJI [19] stated serum CRP and ESR should always be performed in patients with suspected PJI, but low CRP and ESR cannot rule out PJI, the meaning of various new serological markers has also been checked in PJI diagnosis [5–13]. One of these tested serological markers, D-Dimer, which was traditionally used for venous thromboembolism (VTE) detecting, recently has been demonstrated as a promising marker and to perform better than CRP and ESR in PJI diagnosis [12, 13]. However, in this study, we found that D-Dimer does not perform better than CRP and ESR in PJI diagnosis. From our own perspective, the underlying reasons may be as follows: (1) D-Dimers are fibrin degradation products formed due to fibrin clot dissolution by plasmin. Elevated D-Dimer not only be observed in deep vein thrombosis or pulmonary embolism, but also in inflammation, surgery, cancer, infection, injuries, hemorrhages, and many others [20]. So, in theory, it is not a specific marker for the distinction between PJI and aseptic loosening. (2) Although D-Dimer has been demonstrated to rise in septic arthritis, Ribera et al. [21] showed that it is synovial D-Dimer other than serum D-Dimer which is elevated in vivo study of foals with septic arthritis. (3) In Yong et al.’s paper [12], they emphasized that D-Dimer’s role is only effective in early (less than 6 weeks after operation) PJI diagnosis with the combination of the ESR and CRP. However, in our study, the interval between previous joint arthroplasty and admission to our hospital in PJI and aseptic loosening patients is more than 3 months. (4) In Alisina et al.’s paper [13], they found the sensitivity and specificity of D-Dimer in PJI diagnosis is better than CRP and ESR. However, in this study, 11 of 23 aseptic loosening patients (11/23, 47.83%) have serum D-Dimer more than 0.85 μg/L and 3 of 8 low-virulent PJI patients (3/8, 37.5%) have serum D-Dimer less than 0.85 μg/L, which means D-Dimer cannot be used to distinguish between PJI and aseptic loosening. But due to the limited number of our samples, we need more samples to further support our conclusion.

However, we still think our conclusion is more reliable than Alisina et al. [13]. Different from Alisina et al.’s study, we excluded patients with systemic inflammation such as rheumatoid arthritis, psoriasis, SLE, polymyalgia rheumatica, hepatitis B and C, IBD, sarcoidosis, gout, myelodysplastic syndrome, lymphocytic leukemia, and multiple myeloma, as these conditions may induce high expression of CRP, ESR, and D-Dimer [22], which could result in high false positive rates. Of course, there are also a few limitations in our study: 1, the number of patients in our study is only 101, much lesser than Alisina et al.’s study. So, in order to make our conclusion more reliable, we need more samples; 2, as patients with a history of recent dislocation or trauma (within 2 weeks) were excluded in our study, there were difference when compared proportion of knee and hip arthroplasty among three groups, which decreased the reliability of our conclusion to some extent; 3, in this study, we exclude patients with systemic inflammation which account for almost 15% patients in our department; as a result, our conclusion has limitations in clinical utilization.

Overall, in this study, we found serum D-Dimer was highly expressed in PJI and aseptic patients than control patients, which indicates that patients with high level of D-Dimer after joint arthroplasty especially those with uncomfortable symptoms such as pain and fever should be carefully followed up as they may be suffered with PJI or aseptic loosening. Although, in 2018, Javad et al. [23] set serum D-Dimer as one of the criteria for PJI diagnosis, no further data were provided to support the meaning of D-Dimer in PJI diagnosis. So, without further data, at least based on our conclusion, we cannot say D-Dimer is better than CRP and ESR in PJI diagnosis.

## Conclusion

D-Dimer is not a parameter to distinguish between aseptic loosening and PJI.

## Data Availability

The data and materials are available from the department of Orthopedics, Henan Provincial People’s Hospital. The datasets used and analyzed during the current study are available from the corresponding author on reasonable request. The datasets used and analyzed during the current study are available from the corresponding author on reasonable request.

## Abbreviations

CRP: C-reactive protein
ESR: Erythrocyte sedimentation rate
PJI: Prosthetic joint infection
